# A Case of Subacute Degeneration of the Spinal Cord Due to Vitamin B12 Deficiency Triggered by Nitrous Oxide Use

**DOI:** 10.7759/cureus.48312

**Published:** 2023-11-05

**Authors:** Alvin Soh Weng Yan, Abuobeida Ali, Jordon Kong, Lewis Cooney, Junaid Akhtar, Tina Maheswaran, Michael Davies, Yash Prasad

**Affiliations:** 1 Internal Medicine, Countess of Chester Hospital NHS Foundation Trust, Chester, GBR; 2 Gastroenterology, Peterborough City Hospital, Peterborough, GBR; 3 Radiology, Countess of Chester Hospital NHS Foundation Trust, Chester, GBR; 4 Gastroenterology and Hepatology, Countess of Chester Hospital NHS Foundation Trust, Chester, GBR

**Keywords:** peripheral neuropathy, nitrous oxide inhalation, nitrous oxide myelopathy, rare cause of vitamin b12 deficiency, subacute combined degeneration of the spinal cord, nitrous oxide toxicity

## Abstract

In this case report, we discuss a young female who presented to the emergency department with a recent onset of weakness, paraesthesia, and gait disturbance suggestive of peripheral neuropathy and gait ataxia. This was attributed to the occasional use of recreational nitrous oxide (N2O) over the past 10 months. Subacute combined degeneration of the spinal cord is a condition affecting the lateral and posterior columns of the spinal cord, mainly caused by demyelination. The use of recreational N2O depletes the levels of vitamin B12 thus leading to this demyelination of the nervous system. Physical examination revealed T6 and T7 and L3 and L4 sensory deficits bilaterally with hyporeflexia in bilateral knee and ankle reflexes with reduced power in the left lower limb as well as a spastic gait. Her vitamin B12 levels were low (98 g/dL). MRI spine showed a high signal in the posterior cord/ dorsal column. The patient made good recovery post-intramuscular B12 administration and physiotherapy with planned outpatient neurology rehabilitation.

## Introduction

Subacute combined degeneration of the spinal cord is a neurological complication characterised by the effects of vitamin B12 deficiency. It is a demyelination of the dorsal and lateral columns of the spinal cord. Vitamin B12 deficiency can be mainly caused by a few factors involving low vitamin B12 intake, autoimmune causes such as pernicious anaemia and Sjögren’s syndrome, surgery such as postgastrectomy, malabsorption such as Crohn’s disease or coeliac disease, and drug consumption such as metformin, proton pump inhibitors and histamine H2-receptor antagonist [1].

Another cause of low vitamin B12 levels in the body can be due to recreational nitrous oxide use. Patients with neurological manifestation of low vitamin B12 levels can usually present with limb weaknesses, paraesthesia, sensory deficits, and gait disturbances [2]. Here, we report a case of a young female who presents to the emergency department (ED) with signs and symptoms of peripheral neuropathy. From further history taking, blood tests and imaging, we concluded that she developed a subacute degeneration of the spinal cord secondary to vitamin B12 deficiency that was likely caused by nitrous oxide use. She eventually made gradual recovery postadministration of vitamin B12 intramuscularly and physiotherapy.

## Case presentation

A 28-year-old female presented to the ED with a sudden onset of bilateral lower limb weakness with sensation of pins and needles in both her lower limbs. She presented to the ED with worsening weakness for the past five days in her lower limbs. She had no previous history of any medical conditions. With regard to her social history, she had been an occasional N2O user where she inhales the equivalent of one large N2O canister per week which amounts to approximately 640 grams. She had been using this for the past 10 months prior to her admission.

She reported symptoms of lower limb paraesthesia as well as sensory deficits on her feet bilaterally which occurred two days after experiencing the weakness. On neurological examination, the patient had normal cognition and cranial nerves were intact. Upon assessment, power was normal with 5/5 in all four limbs with hyporeflexia seen in knees and ankles bilaterally. Tone was otherwise normal and on soft touch, there is the presence of T6 and T7 and L3 and L4 sensory deficits bilaterally. Her coordination was grossly normal and she displayed a spastic gait. 

Investigations 

With regard to investigations, her full blood count was normal with no signs of anaemia. Her blood investigations also showed that her vitamin B12 levels were dangerously low (98). Upon further investigation of this B12 deficiency, homocysteine and methylmalonate were raised at 58.0 and 3.02 respectively. A full autoimmune serology workup was performed which was normal. Table 1 summarises all the blood investigations that were done during the patient’s admission.

**Table 1:** Blood investigations on admission including a full autoimmune workup

Blood tests	Results	Reference values	Units
Haemoglobin	127	115-160	g/L
White cell count	4.2	4.0-11.0	x10^9/L
Platelet	317	120-400	x10^9/L
Partial Thromboplastin Time	26.5	26.0-35.0	seconds
Prothrombin Time	14.1	12.5-15.0	seconds
Vitamin B12	100 (L)	150-750	ng/L
Folate	12.1	4.0-50.0	ug/L
Sodium	137	133-144	mmol/L
Potassium	3.5	3.5-5.0	mmol/L
Urea	4.3	2.0-6.5	mmol/L
Bicarbonate	30	20-32	mmol/L
Creatinine	59	50-120	umol/L
Bilirubin	17	3-20.0	umol/L
Alanine Transaminase	58 (H)	8-45	U/L
Albumin	35	32-46	g/L
Total protein	69	60-80	g/L
Alkaline Phosphatase	96	46-148	U/L
Creatine kinase	279 (H)	26-140	U/L
Magnesium	1	0.70-1.0	mmol/L
Phosphate	0.98	0.80-1.40	mmol/L
Thyroid Stimulating Hormone	1.29	0.35-5.50	mU/L
Free T4	13.9	7.86-14.41	mU/L
Cortisol	239	150-650	nmol/L
Serum copper	23.9	11.0-25.0	umol/L
Serum caeruloplasmin	0.39	0.15-0.47	g/L
Serum homocysteine	58.0 (H)	5.0-14.0	umol/L
Methylmalonate	3.02 (H)	0.00-.028	umol/L
Perinuclear anti-neutrophil Cytoplasmic Antibodies (pANCA)	Negative		
Antineutrophil Cytoplasmic Autoantibody (cANCA)	Negative		
GM1GG Antibodies	Negative		
GM1 IgM	Negative		
IgG Antibodies to Glycolipid GM1	Negative		
IgM Antibodies to Glycolipid GM1	Negative		
IgG Antibodies to Glycolipid GQ1B	Negative		
IgM Antibodies to Glycolipid GQ1B	Negative		
Anti-gastric parietal cells antibodies	Negative		

**Figure 1:**
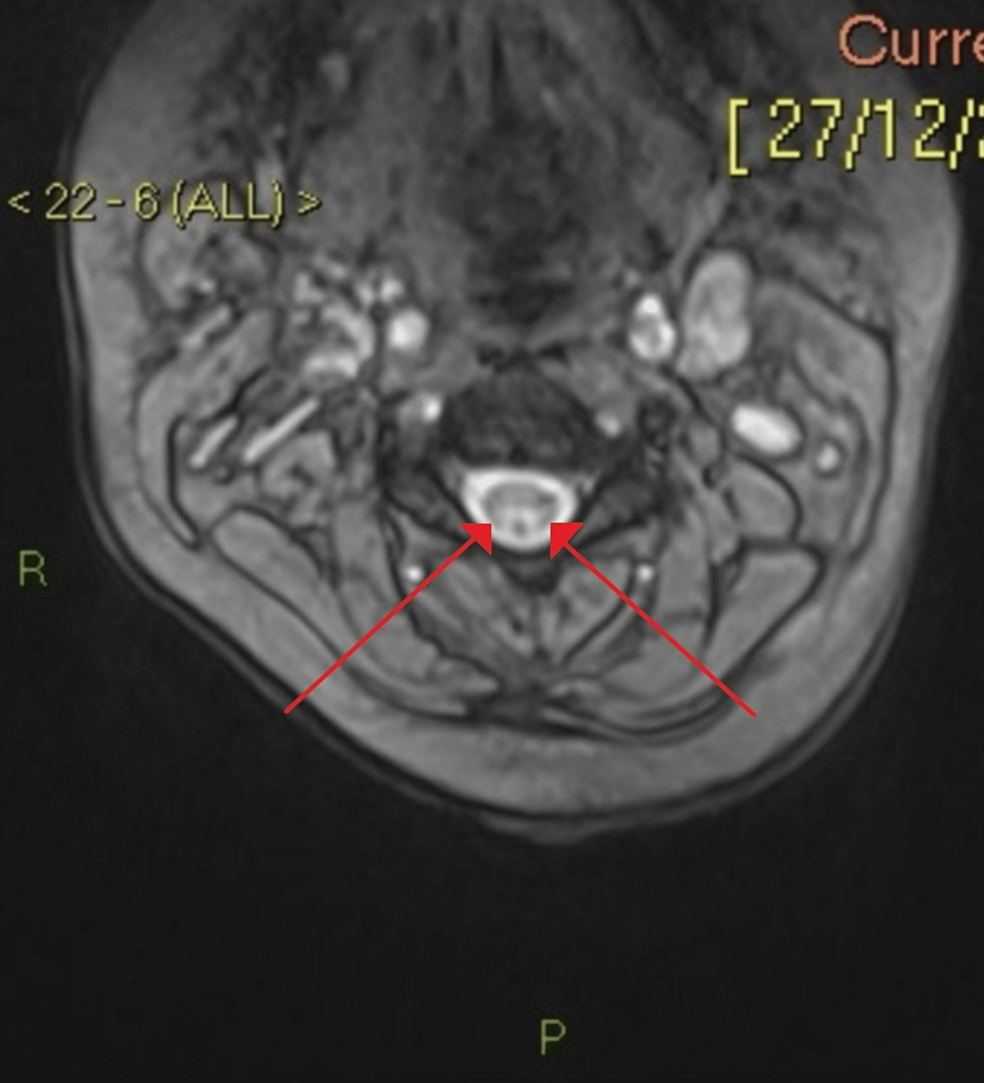
MRI spine demonstrating symmetrical high signalling in the dorsal column/posterior spinal cord from C2-C6

MRI whole spine demonstrated T2/STIR signal hyperintensity of the cervical and upper thoracic cord as shown in Figure 1. This showed a predilection of the posterior cord/dorsal columns which is typical of combined subacute degeneration of the spinal cord. There was no enhancement following the administration of contrast. 

Differential diagnosis

The differential diagnoses that we considered in this instance included diseases of an autoimmune aetiology (Guillain-Barre syndrome or autoimmune myelopathy). Demyelinating diseases such as multiple sclerosis were also considered; however, in this case it was unlikely given the characteristics of her symptoms which had not fulfilled the McDonald’s criteria for multiple sclerosis and MRI findings were atypical. Paraneoplastic syndrome was also considered however her presentation was not characteristic of this, with a low likelihood of neoplasm and no cognitive symptoms including speech, memory or visual changes. 

Nutritional deficiencies such as B12 deficiency and copper deficiency were also considered. History was not suggestive of a case of pernicious anaemia. Serum copper was investigated which was normal in this case. By exclusion of other differentials, in addition to low serum vitamin B12 levels, we investigated this deficiency further. Ultimately, given her history, clinical presentation, low serum vitamin B12 levels and findings of myelopathy from her MRI spinal cord, we concluded that this was the most likely diagnosis.

Treatment

Following this diagnosis, she was subsequently treated with intramuscular (IM) hydroxocobalamin injections of 1000 milligrams daily for a week, weekly for a month and monthly until improvements stopped. Clinical improvements were observed on the ward following the start of treatment with a decrease in her lower limb weakness as well as improvement in her sensory deficits. She eventually made a gradual recovery after two weeks with a combination of IM vitamin B12 administration and physiotherapy. She was seen by the neurology team and deemed no further follow-up was necessary from a neurology point of view. She had a telephone consultation and the patient confirmed almost complete resolution of her symptoms 10 months after discharge.

## Discussion

Nitrous oxide (N2O) also known colloquially as ‘laughing gas’ is a colourless gas which has been used as an anaesthetic and analgesic in medical/ dental settings [3], as well as for industrial and commercial settings. Recreational N2O use is prevalent among younger people for its euphoric effects and inducing feelings of relaxation and calmness. There is a reported 2%-15.8% lifetime prevalence of nitrous oxide abuse among teenagers and adults of a younger population [4]. In addition to this, according to the Office for National Statistics, N2O was the third most abused drug, after cannabis and cocaine, in 2020. 

Vitamin B12 deficiency is the cause of subacute combined degeneration [5]. The mechanism occurs through the inactivation of vitamin B12 by N2O which converts its monoactive form to an inactive bivalent form [6]. Vitamin B12, in this instance, is important for its function as a cofactor in the synthesis of methionine synthase which catalyzes the synthesis of methionine into S-adenosylmethionine (SAM). SAM is involved in the production of phospholipids which are the primary building blocks of myelin [7]. Through this physiological process, we find that the inhalation of NO2 depletes the levels of vitamin B12 which causes the demyelination of the nervous system, including the spinal cord and peripheral neuropathy. With this effect on the nervous system, patients abusing N2O use can present clinically with subacute onset of paraesthesia and paraparesis. History taking and identifying key events and symptoms are very important in identifying these cases. Clinical findings may also include serum vitamin B12 levels that are low during admission, in addition to symmetrical T2 hyperintensity of the dorsal columns of the cervical cord which is in keeping with symptoms suggestive of peripheral neuropathy. Methylmalonate levels can also be used to support the diagnosis in the event of a normal vitamin B12 level [8]. 

General treatment would be administering intramuscular vitamin B12 injection to replace the ongoing deficit and in this case, it is recommended to stop recreational N2O use. This plus ongoing physiotherapy appears to be the gold standard in general treatment of subacute degeneration of the spinal cord triggered by NO2 use. 

The indicators for prognosis for a patient with a subacute combined degeneration of the spinal cord due to Vit B12 deficiency remains unknown [9]. Administration of vitamin B12 does lead to improvement of neurological function however only a few patients report complete recovery. Studies suggest that an absence of sensory level, Romberg and Babinski’s signs as well age being less than 50 years old are associated with a higher rate of clinical resolution for signs and symptoms of a patient with subacute combined degeneration of the spinal cord [9,10]. This is not surprising given that the presence of a positive Romberg’s sign is attributed to compromise on the functioning of the dorsal column in the spinal cord, whereas the presence of Babinski’s sign suggests lateral corticospinal tract dysfunction [10]. Further literature also suggests that age is a factor in the prognosis of a patient with a vitamin B12 deficiency-induced subacute degeneration of the spinal cord. The age of patients <50 is reported to have a better prognosis given a better nervous system plasticity [9].

There is a rising number of neurological complications that arise due to misuse of nitrous oxide [11]. The UK government has recently decided to place a ban on the misuse of nitrous oxide citing antisocial behaviour, increase in use and ‘growing harm’ as major keys in driving the decision to place the ban [12]. This decision has received several criticisms from experts as the decision to place the ban on recreational use of nitrous oxide was deemed as not being evidence-based [13]. There is a reported mortality rate of 1 in 1 million nitrous oxide users in the UK per year [13]. The arguments behind the ban on the recreational use of nitrous oxide is because with this ban being in place, those that abuse it will now move on from the usual canisters to cylinders which have a higher dosing henceforth causing a potential for more severe complications due to its misuse [13]. A reported survey from 2019-2020 found that 9% of those aged between 16 and 24 years old had taken the drug, a stark increase from a reported 6.1% back in 2012-2013 [11]. For the age groups reported in the survey, it is also the second most popular drug after cannabis [11]. 

Therefore, rather than placing a full-on ban, going down the route of educating its target groups towards the side effects of abuse or misuse of nitrous oxide and its effects towards the body i.e neurological system would perhaps prove to be more futile and beneficial in order to decrease the rate of incidence of nitrous oxide misuse. Due to its increasing incidence rate, especially in younger adults, it is important for clinicians especially to keep an open mind for nitrous oxide abuse, especially as a differential diagnosis for someone who presents with signs and symptoms of peripheral neuropathy. This can be done via a thorough history taking that explores the social history, coupled with several blood tests and imaging to rule out other possible causes of peripheral neuropathy as described in this case report.

## Conclusions

With the increasing number of incidence rates and rising neurological complications as well as the recent ban towards the recreational use of nitrous oxide, it is important to keep an open mind regarding the diagnosis of subacute combined degeneration of the spinal cord due to vitamin B12 deficiency as well as other causes of peripheral neuropathy. Thorough history taking, mainly exploring the social context of the patient as well as images viewed from MRI will ensure that the patient receives appropriate treatments in due time. Treatments such as B12 supplementation and physiotherapy with neurological rehab have been proven to achieve clinical resolution in patients.
